# Results of the Insertion of Hysteroscopic Sterilization Devices in a Brazilian Public Hospital

**DOI:** 10.1055/s-0040-1712129

**Published:** 2020-06

**Authors:** Daniele Lauriano Pastore, Luiz Guilherme Pessoa da Silva, Ricardo Bassil Lasmar

**Affiliations:** 1 Department of General Surgery ad Specialty, Universidade Federal Fluminense, Niterói, RJ, Brazil; 2 Department of Obstetrics, Hospital da Mulher Mariska Ribeiro, Rio de Janeiro, RJ, Brazil

**Keywords:** tubal sterilization, hysteroscopy, female sterilization, contraceptive devices, esterilização tubária, histeroscopia, saúde da mulher, dispositivos anticoncepcionais

## Abstract

**Objective** To evaluate the insertion of the hysteroscopic intratubal sterilization device for female sterilization concerning the technique and the feasibility.

**Methods** Retrospective study with data collection of medical records of 904 patients who underwent device insertion between January and September 2016 in a public hospital in Rio de Janeiro (Brazil) with data analysis and descriptive statistics.

**Results** In 85.8% of the cases, the uterine cavity was normal, and the most commonly-described findings upon hysteroscopy were synechiae (9.5%). The procedure lasted an average of 3.56 minutes (range: 1 to 10 minutes), and the pain was considered inexistent or mild in 58,6% of the cases, mild or moderate in 32,8%, and severe or agonizing in less than 1% (0.8%) of the cases, based on a verbal scale ranging from 0 to 10. The rate of successful insertions was of 85.0%, and successful tubal placement was achieved in 99.5% of the cases. There were no severe complications related to the procedure, but transient vasovagal reactions occurred in 5 women (0.6%).

**Conclusion** Female sterilization performed by hysteroscopy is a safe, feasible, fast, and well-tolerated procedure. The rates of successful insertions and tubal placements were high. There were few and mild adverse effects during the procedure, and there were no severe complications on the short term.

## Resumo

**Objetivo** Avaliar a inserção de dispositivo intratubário de esterilização histeroscópica com relação à viabilidade e à técnica.

**Métodos** Estudo retrospectivo com coleta de dados de prontuários das pacientes submetidas à inserção do dispositivo entre janeiro e setembro de 2016 em um hospital público do Rio de Janeiro, com análise dos dados e realização de estatísticas descritivas.

**Resultados** Foram incluídos 904 casos no estudo. Em 85,8% dos casos, a cavidade uterina estava normal, e os achados mais comumente descritos à histeroscopia foram as sinequias (9,5%). O tempo médio do procedimento foi de 3,56 minutos (gama: de 1 a 10 minutos); a dor foi considerada de ausente a leve em 58,6% dos casos, de leve a moderada em 32,8% dos casos, e de forte à pior dor possível em menos de 1% dos casos (0,8%). A taxa de inserções bem-sucedidas foi de 85,0%, e a colocação tubária foi bem-sucedida em 99,5% dos casos. Não foram identificadas complicações graves, mas reações vasovagais transitórias ocorreram em 5 mulheres (0,6%).

**Conclusão** A esterilização feminina por histeroscopia é um procedimento seguro, viável, rápido, e bem tolerado. As taxas de inserção bem-sucedida e de colocação tubária foram altas. Houve poucos e leves efeitos colaterais durante o procedimento, e não foram observadas complicações graves no curto prazo.

## Introduction

For a few decades, laparoscopic tubal ligation has been considered the reference procedure for female sterilization. Although it is a safe method, it is associated with rare complications related to general anesthesia, or vascular or organ damage.^1^

The hysteroscopic intratubal sterilization device (Essure, Conceptus, Inc., Mountain View, CA, US) was developed with the intention of promoting an outpatient sterilization method without the need for general anesthesia, without abdominal incision, which enables a faster return to activities. It is a 4-cm long, 0.8 mm in diameter, spring-shaped microimplant, internally composed of stainless steel and polyethylene terephthalate (PET) fibers and externally coated with a nitinol (nickel titanium) alloy.^2^ ^3^ ^4^ ^5^

The device initially appears in a compressed form, and is inserted by hysteroscopy to reach the proximal fallopian tube under direct vision. After being positioned, there is an expansion of the implant that anchors it to the tube, reaching 2 mm in diameter. Polyethylene terephthalate fibers stimulate the proliferation of adjacent tissue through inflammation and the formation of fibrosis enveloping and infiltrating the device. The result is tubal occlusion after a few weeks.

After the approval of the device as a hysteroscopic sterilization method by the Brazilian Health Regulatory Agency (Agência de Vigilância Sanitária – Anvisa, in Portuguese), it was implemented as the choice for sterilization at Mariska Ribeiro Women's Hospital (Hospital da Mulher Mariska Ribeiro, HMMR, in Portuguese), a municipal hospital located in Rio de Janeiro, which became the only public hospital in the city to perform this procedure within the Brazilian Unified Health System (Sistema Único de Saúde – SUS, in Portuguese).

Despite encouraging results published in the literature on the safety and efficacy of the device and patient satisfaction, the product was largely criticized due to its association with serious adverse effects that led to surgical interventions for its removal.^3^ ^5^ ^6^ The major complaints included pelvic pain, bleeding, and edema. In February 2016, the US Food and Drug Administration (FDA) inserted a warning on the label of the product to advise the patients about the complications associated with this device, and ordered the company to conduct a postmarket surveillance study including at least 2,000 women for 3 years, comparing the efficacy and the safety of the device to the other sterilization methods. This atmosphere of complaints and insecurity culminated in the removal of the product from the market in December 2018 in the US.

In parallel with the history of intrauterine device (IUD) contraception, it is possible that threatening information to the adverse event method without proper scientific backing is only a temporary scenario. In this context, the scientific technical reinforcement with more research related to the topic worldwide becomes important to evaluate the method as a safe technique for female sterilization.

The present study aimed to retrospectively evaluate the results of the insertion of the hysteroscopic intratubal sterilization device in a Brazilian public hospital, regarding the insertion technique and the patient's tolerability.

## Methods

The present is a retrospective study with analysis of medical records of a convenience sample. We included data from the medical records of patients who underwent insertion of the hysteroscopic intratubal sterilization device at HMMR, from January to September 2016. The information contained in the medical records until December 2016 were considered for the study.

All medical records of patients submitted to the insertion of the device at HMMR during the described period were recruited. The population included women of reproductive age, who showed interest in sterilization at Basic Health Units (BHUs) in Rio de Janeiro and were referred to HMMR between January and September 2016, with more than 2 children or older than 25 years of age, meeting the criteria of the Brazilian law regarding voluntary sterilization, and who agreed with the hysteroscopic tubal occlusion as a sterilization method. There was no loss of cases, as well as no selection control of the participating patients.

The exclusion criteria for the procedure were: uncertainty about choosing a permanent method; insecurity regarding the method of tubal obstruction; patients who had delivered in the previous 6 weeks, or who terminated the pregnancy in the second trimester; patients with active or recent gynecological infections; those with suspected or confirmed gynecological malignant tumors; patients with proven allergy to nickel; those with positive β-hCG at the date of the insertion; and patients with systolic blood pressure higher than 160 mmHg and/or diastolic blood pressure higher than 90 mmHg at the time of the examination.

On the day of the procedure, the vital signs of the patient were evaluated, the β-hCG was collected, and a summary anamnesis was performed. All patients received 10 mg of diazepam and 600 mg of ibuprofen orally 30 to 60 minutes before the insertion of the device. The procedures were performed an a day hospital by 3 professionals in hysteroscopy with more than 8 years of experience.

The hysteroscopy technique used was vaginoscopy, with a 30° angled lens Bettocchi (Karl Storz SE & Co. KG, Tuttlingen, Germany) 5-Fr hysteroscope with 2.9 mm in diameter. The patient was placed in the lithotomy position and the distention medium was saline solution, which was hung at a height of ∼ 1 m above the patient, enabling an average infusion pressure of 80 mmHg to be obtained.

The insertions were performed with a 1.7-mm catheter containing the device. It was positioned in the isthmus of the fallopian tube, bilaterally, reaching its 2-mm diameter. Positioning was considered adequate when 3 to 8 turns of the device remained visible in the uterine cavity immediately after the insertion. The procedure time was measured in minutes, with the physician already positioned, including the hysteroscopy, the insertion of the devices, and the removal of the instruments from the vaginal canal. Pain was assessed using a verbal scale from 0 to 10, with 0 meaning “no pain” and 10, the “worst possible pain.” After the hysteroscopy, the patients were sent to a room to rest, and the adverse effects were objectively recorded in a specific form: cramps (mild, moderate or severe); nausea (yes or no); and vomiting (yes or no). Hypotension was recorded descriptively.

Three months after the insertion, the patients returned for a transvaginal ultrasound, in which it was possible to identify the correct positioning of the device and infer the tubal occlusion, which suggests definitive sterility. If there was doubt about positioning, a new hysteroscopy or pelvic radiograph could be performed. Only after the performance of hysterosalpingography (HSG), which began in the hospital in July 2016, the patients underwent this confirmatory examination in cases of doubtful transvaginal ultrasound or unilateral device insertion.

Hysterosalpingography was indicated in cases of unilateral insertion due to impossibility of insertion in the other side because of suspicion of tubal obstruction. If bilateral tubal obstruction was confirmed, sterilization was assured, and the patient was discharged from follow-up. In case of failure to insert the device in both sides, but not confirmed on hysterosalpingography, the patient was referred to the BHU to undergo tubal ligation or correction of the uterine pathology presented. The insertion, therefore, can be divided according to the following possibilities: successful insertion in the first attempt (bilateral first-attempt insertion or unilateral insertion in patients with contralateral salpingectomy); successful bilateral insertion in the second attempt; unilateral insertion failure; and bilateral insertion failure.

The information was recorded in a database using the Excel (Microsoft Corp., Redmond WA, US) software, version 2013, and the statistical analysis was then performed using the Statistical Package for the Social Sciences (SPSS, IBM Corp. Armonk, NY, US) software, version 22.0. The variables analyzed were age, marital status, number of pregnancies, parity, mode of delivery, length of the procedure, pain during the procedure, technical difficulty to insert the device, the need for maneuvers, the immediate and late complications after the procedure, evaluating only the first 3 months of insertion. Descriptive statistics were performed, including average, percentage and confidence interval (CI), as well as the Shapiro Wilk normality test for data evaluation.

The patient's' confidentiality was preserved, and access to the information on the medical record was reserved exclusively for those involved in the research and the institution's health team. The study was approved by the Brazilian Ethics Committee (Comitê de Ética e Pesquisa, CEP, in Portuguese) under CAAE number 56703216.0.0000.5275. There were no conflicts of interest.

## Results

In the present study, 904 medical records were reviewed. The demographic characteristics of the sample can be seen in Table 1.

**Table 1:** Characteristics of the patients who underwent insertion of the Essure intratubal device

Clinical data	n(%)	95% confidence interval
*Age (years)*	32.3(18–49)	31.9–32.6
*Marital status*		
Single	595(65.8)	
Stable union	276(30.5)	
Divorced	23(2.5)	
Widow	2(0.2)	
No record	8(0.9)	
*Pregnancies*	3.3 (1–12)	3.2–3.4
*Deliveries*		
Normal delivery	2.2 (0–11)	2.1–2.3
Cesarean section	0.66 (0–7)	0.60–0.72
*Abortion*	0.42 (0–10)	0.36–0.47

At the time of the insertion of the device, in 893 cases (98.8%) there was no need for maneuvers; in 10 cases (1.1%), lysis of the peripheral synechiae was performed; and in 1 case (0.1%), dilation of one ostium was performed. The anatomical findings on hysteroscopy were summarized in Table 2.

**Table 2:** Distribution of the anatomical findings described on the hysteroscopy of the patients who underwent insertion of the Essure intratubal device

Anatomical findings	n(%)
No findings	776(85.8)
Synechiae	86(9.5)
Endometrial polyp	15(1.7)
Submucosal myoma	9(1.0)
Endometritis	9(1.0)
Ovular remains	2(0.2)
Mullerian anomalies	2(0.2)
Endometrium hypertrophy	2(0.2)
Periorificial hyperemia	1(0.1)
Adenomyosis	1(0.1)
Total	904(100.0)

In only 1 case (0.1%), the endometrial cavity could not be accessed due to significant cervical stenosis. The distribution of the insertion outcomes (unilateral or bilateral) and the confirmation by imaging are summarized in Table 3. The cases of asymptomatic endometritis seen on hysteroscopy were treated with antibiotic therapy, and there was no contraindication to the insertion in these cases.

**Table 3:** Distribution of outcomes regarding the insertion of the Essure intratubal device

Outcomes	n(%)
**First-attempt bilateral insertion**	766(84.7)
Confirmed by image	762(84.2)
Waiting for hysterosalpingography	3(0.3)
Lost to follow-up	1(0.1)
**First-attempt unilateral insertion**	29(3.0)
Bilateral insertion on second attempt	9(1.0)
Bilateral tubal occlusion	7(8.0)
Unilateral tubal occlusion	3(0.3)
Waiting for hysterosalpingography	8(0.9)
Unilateral salpingectomy	2(0.2)
**No insertion**	109(12.0)
Bilateral occlusion	99(11.0)
Cervical stenosis	1(0.1)
Treatable disorders	9(1.0)

**Fig. 1:**
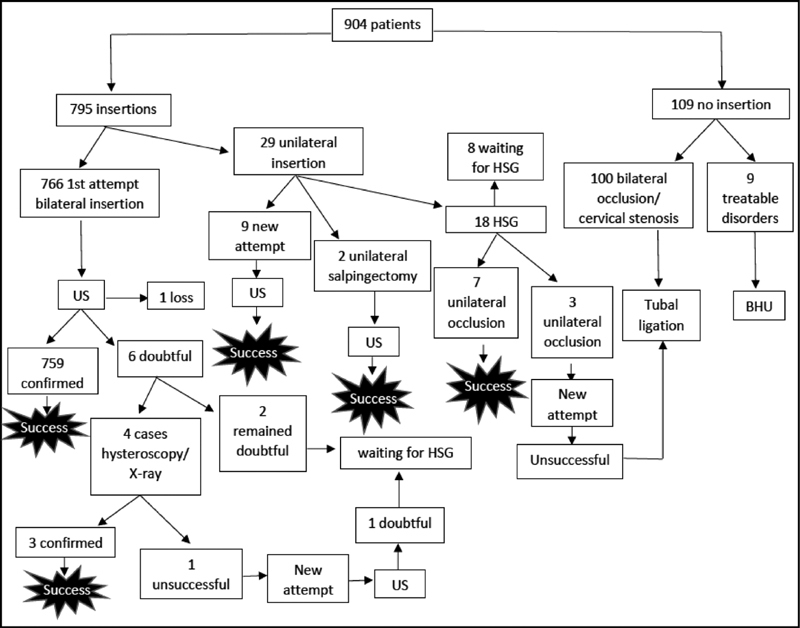
Essure insertion flowchart.

In 1 case (0.1%), there was no view of the device on one side, and the hysteroscopy showed the other device in the endometrial cavity: this was considered the only case in which a device was expelled. Fig. 1 shows the follow-up and management flow chart for all patients analyzed. The cases considered successful were those in which the bilateral insertion of the devices was completed. The three cases that were waiting for HSG were lost to follow-up after the study (Fig. 1).

**Fig. 2:**
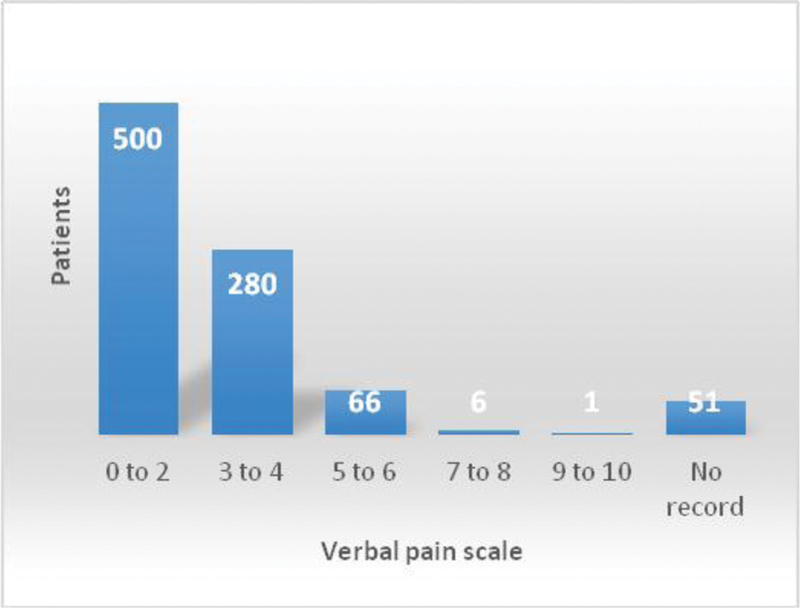
Column chart showing the distribution of the patients according to the pain assessed by the verbal pain scale at the time of insertion of the Essure intratubal device.

The procedure time ranged from 1 to 10 minutes, with an average of 3.56 minutes (95%CI: 3.47–3.63), Shapiro Wilk 0 normality test, considering the record of 856 medical records. The data on pain reported during the procedure were obtained from 853 medical records. The pain variation according to the verbal scale was from 0 to 9, with an average of 2.18 (95%CI: 2.07–2.28), and the pain was assessed using a verbal scale from 0 to 10, with 0 meaning “no pain”, and 10, the “worst possible pain.” Fig. 2 shows the distribution of pain in the sample.

The distribution of symptoms presented after the immediate procedure can be seen in Table 4.

**Table 4:** Distribution of complications presented in the immediate postoperative period of patients submitted to the insertion of the Essure intratubal device

Variable	n(%)
*Cramps*	
No	50(5.5)
Mild	720(79.6)
Moderate	88(9.7)
Intense	46(5.1)
*Nausea*	
No	872(96.5)
Yes	32(3.5)
*Vomit*	
No	884(97.8)
Yes	20(2.2)
*Hypotension*	
No	899(99.4)
Yes	5(0.6)

Within 3 months of follow-up, only 2 patients (0.2%) had any complaints: 1 case of abnormal uterine bleeding (0.1%), and 1 case of leukorrhea (0.1%) with a fetid odor.

A successful insertion rate can be considered as the sum of the cases in which bilateral insertion was successful in the first attempt (766 cases), as well as of those of successful unilateral insertion in patients previously submitted to contralateral salpingectomy (2 cases) over the total number of patients (904 cases), resulting in a rate of 85.0% in the present study. The insertion success rate, in turn, can be obtained by the ratio between the number of cases confirmed by imaging examination 3 months after successful insertion in the first attempt (764 cases) and the total number of successful insertions in the first attempt (768 cases), and it was of 99.5% in the present study.

As a study based on medical records, it took 3 months of follow-up, but some of the information needed for the analysis were incomplete, impairing some results. Although establishing a pregnancy rate is not the objective of the present study, there were no ongoing pregnancies registered on the medical records at the time of the analysis.

## Discussion

Data from the medical records of 904 patients were analyzed. There was an average of 3.3 pregnancies and 2.9 deliveries per patient, with a sample similar to that presented by Depes et al.^7^ The average age of the patients was 32.3 years (95%CI: 31.9–32.6), which was similar to the average of the study by Depes et al^7^ (34.5 years), representing a sample of young patients. In similar studies conducted in Europe, the average age was higher, such as those presented by Franchini et al^8^ (39.5 years), in a study conducted in Italy, and by Fernandez et al^9^ (41 years), in a study conducted in France. This age difference is possibly explained by socioeconomic factors, since the average age at the first pregnancy in countries with lower socioeconomic development is usually lower than in developed countries.

The procedure was performed under the day-hospital regime. Studies show that the effectiveness of the insertion of the device in this model is similar to that of procedures performed in the operating room.^6^ The patients in our study received 10 mg of diazepam and 600 mg of ibuprofen orally 30 to 60 minutes before the procedure, which is in line with the conduct adopted by most services.^1^ ^3^ ^5^ ^7^ ^9^ The prescription of anti-inflammatory and antispasmodic drugs was justified in these studies in order to prevent tubal spasm.

No anesthetic was used for the procedure, following the most commonly proposed technique. According to Bernardo and Vázquez-Carmino,^10^ anesthesia, whether local or general, does not increase the safety and the success of the method, does not improve the hospital recovery time, increases the exclusion criteria, decreases the inclusion criteria, considering the contraindications related to it, and increases the cost, lowering the profitability of the method.

Out of the 109 cases without insertion, 99 cases were due to bilateral obstruction observed during the examination, representing 11% of all patients. These patients were referred directly to the BHU for tubal ligation without a new attempt at insertion. Part of these cases was probably due to tubal spasm, and part was possibly due to tubal obstruction due to a past salpingitis, which was not evaluated in our study. A study by Leyser-Whalen et al^11^ showed a positive correlation between insertion failure and history of sexually-transmitted diseases. It is still an initial study, with limitations, but correlating these two factors is quite reasonable.^11^

Device insertion was fast in the present study, with an average of 3.56 minutes (95%CI: 3.47–3.63) for bilateral insertions, a result similar to that presented by Depes et al^7^ (4.5 minutes), but much shorter than the average presented by Chern and Siow^3^ (22.8 minutes). This disparity can be explained by the difference between the techniques used. In Chern and Siow's^3^ study, vulvar antisepsis, vaginal speculum insertion, and vagina and cervix antisepsis were performed with the same solution. In most studies, including ours, however, the vaginoscopy technique introduced by Bettocchi and Salvaggi^12^ in 1997 is preferred because it is considered faster and less uncomfortable for the patient, as already established in the literature.^13^ Performing antisepsis led to increased procedure time and did not appear to be beneficial, as there were no cases of complications related to infection within 3 months in our study. The time difference could also be explained by an exposure to the initial experience of using this device in a service in Singapore in the study by Chern and Siow.^3^ Although the present is a study with data on insertions performed in 2016, our hysteroscopy professionals have been performing insertions since 2014.

Pain during the procedure was assessed by the verbal scale, with an average of 2.18 in our study, which is very close to the value presented by Depes et al^7^ (3.0). However, we did not use the Visual Analog Scale (VAS), which could bring some difference due to the higher subjectivity. A result such as the one herein presented is equivalent to mild pain, and means, therefore, that the examination was well-tolerated by the patient. Such evidence reinforces the results obtained in the studies by Duffy et al^14^ and Syed et al,^15^ who compared hysteroscopic sterilization with laparoscopic tubal ligation and showed mild pain in the first method and less pain when compared with the second. A fast return to activities and lower cost compared with laparoscopic tubal ligation have also been reported in other studies.^3^ ^16^

There were no serious complications in the present study. Immediately after insertion, while resting in the auxiliary room, mild cramps was the complaint of most patients, present in almost 80% of the cases. Episodes of hypotension were minimal, present in 0.6% of the cases. Such data are close to those presented by Sinha et al.^5^

The successful insertion rate of 85% was compatible with the values presented in previous studies, which present results ranging from 81% to 100%, with variable sample sizes.^7^ ^17^ The 99.5% imaging insertion success rate was consistent with the 90% range found in the review and meta-analysis of La Chapelle et al^17^ including 37 studies regarding this same device. It is important to note, however, that there was a limitation to the analysis of these data, due to the loss of follow-up of one of the cases; the remaining three included cases were still awaiting HSG.

Several studies defend the use of transvaginal ultrasound to evaluate the device positioning as a preferred imaging method, as it avoids radiological exposure, it is more available and more comfortable for the patient when compared with HSG, and it is sufficient to confirm proper positioning in most cases.^18^ ^19^ ^20^ ^21^ ^22^ ^23^ There is no difference in pregnancy rates when comparing the two methods.^24^

In the present study, there were some differences regarding the indication of imaging examination. Transvaginal ultrasound was used as the first examination to evaluate device positioning. However, some cases of unilateral insertion were taken directly to undergo the HSG, and, in cases of doubt regarding the placement of the device, the cases were referred to pelvic radiography, a new hysteroscopy, or HSG. Part of this divergence was due to the lack of available HSG in the beginning of the study. When looking at the world literature, we notice that most European countries use pelvic radiography or transvaginal ultrasound routinely to check the proper positioning of the device, reserving HSG for cases of doubt regarding the correct positioning after the performance of these other viewing modalities. However, in the US, the FDA required HSG as part of the protocol prior to discontinuation of the contraceptive method in use.^25^ Therefore, it is clear the need to create protocols by the services to better systematize and standardize the conducts performed, enabling the optimization of the results found in different countries.

In the last five years, there were many studies reporting the outcomes after hysteroscopic sterilization and comparing to them to the outcomes of laparoscopic sterilization, but still with some conflicting results. Mao et al^26^ made a large observational cohort study to evaluate the outcomes in New York State for over 7 years of 10,109 pairs of women who underwent hysteroscopic and laparoscopic sterilization, and the authors concluded that the estimated risk of undergoing tubal ligation or resection within this time was higher after hysteroscopic sterilization than after laparoscopic sterilization (3,9% versus 1,6% respectively; Hazard Ratio (HR): 2,89; 95%CI: 2,33–3,57). Bouillon et al^27^ made a large cohort study in France including 105,357 women using the national hospital discharge database linked to the health insurance claims database to compare the risk of reported adverse events between the same two groups. The conclusion was that the use of hysteroscopic sterilization was significantly associated with a higher risk of gynecological complications (such as sterilization failure, which includes salpingectomy, second sterilization procedure, or pregnancy and reoperation) over 1 and 3 years when compared to laparoscopic sterilization, but the risk of medical outcomes (including all types of allergies, autoimmune diseases, thyroid disorder, use of analgesics, antimigraine medication, antidepressants, benzodiazepines, outpatient visits, sickness absence, suicide attempts, death) was not significantly increased over 1 or 3 years.

Major studies with long-term follow-up are required for an assessment of the efficacy as well as of the complications associated with the hysteroscopic sterilization method not evaluated in the present study, especially in light of the current criticism and prohibitions regarding the use of the method. Finally, it is extremely important to evaluate the need to preselect the patients and the long-term pregnancy rates, as well as the comparison of costs to the health system in relation to laparoscopic sterilization, which is the current reference procedure.

The present observational study has several limitations, most of them related to the retrospective model. Firstly, as the information was collected from medical records, there was loss of information, which impaired some of the analyses. Secondly, the short time of follow-up did not offer the possibility to evaluate the pregnancy rates, the long-term complications, and the satisfaction of the patients with the method. Thirdly, there was no evaluation of cost and outcomes comparing hysteroscopic and laparoscopic sterilization.

## Conclusion

The insertion of the intratubal device for hysteroscopic sterilization proved feasible, rapid, safe and well-tolerated by the patients. The rate of successful insertions was high. In addition, low short-term complication rates were found, with minor side effects in most cases, and no serious short-term complications were observed. The strength of the present study is due to the large sample size, but bigger studies with a longer follow-up are needed to make a more rigorous evaluation of the efficacy and the associated complications for a stronger scientific support of the technique.
